# Lysophosphatidic acid mediates the pathogenesis of psoriasis by activating keratinocytes through LPAR5

**DOI:** 10.1038/s41392-020-00379-1

**Published:** 2021-01-15

**Authors:** Li Lei, Bei Yan, Panpan Liu, Jie Li, Chao Chen, Wu Zhu, Yehong Kuang, Xiang Chen, Cong Peng

**Affiliations:** 1 The Department of Dermatology, Xiangya Hospital, Central South University, Changsha, China; 2 National Clinical Research Center for Geriatric Disorders, Xiangya Hospital, Changsha, China; 3 Hunan Key Laboratory of Skin Cancer and Psoriasis, Xiangya Hospital, Changsha, China; 4 Hunan Engineering Research Center of Skin Health and Disease, Xiangya Hospital, Changsha, China; 5 Xiangya Clinical Research Center for Cancer Immunotherapy, Central South University, Changsha, China

**Keywords:** Immunological disorders, Immunological disorders

**Dear Editor,**

Psoriasis is a common immune-mediated, chronic inflammatory skin disease, which has been characterized by epidermal acanthosis, hyperkeratosis, parakeratosis and extensive infiltration of inflammatory cell.^1^ KCs have critical roles in skin innate and adaptive immune responses during the development of psoriasis,^1^ which produce large amounts of inflammatory mediators, such as antimicrobial peptides (e.g., S100A8,9), proinflammatory cytokines (e.g., IL-6, IL-17A,C,TNF-α,) and chemokines (e.g., CXCL1,2), triggering innate or adaptive immune responses.^1^

Lipids have essential roles in maintaining normal physiological cellular functions, and there are approximately ten thousands different documented lipids and approximately six hundreds distinct molecular species of human lipids. Lysophosphatidic acid (LPA), the simplest phospholipid found in nature, is a key precursor in the early stage of cellular phospholipid biosynthesis.^2^ Inhibitors or agonists targeting LPA receptors or LPA receptor-deficient mice, demonstrate the diversity of physiological or pathological functions of LPA or LPA receptors. Our previous study found that enzymes involved in glycerophospholipid metabolism, such as LPA, LysoPC and PA, were significantly altered in the plasma of psoriatic patients.^2^ However, the role of LPA and its receptor in the pathogenesis of psoriasis remains elusive.

In this study, we found that LPA levels are significantly increased in psoriatic patients’ serum (Supplementary Fig. 1b) and IMQ-induced mice psoriatic skin lesions (Supplementary Fig. 1a). To study the effect of LPA on psoriasis, we treated human keratinocytes (NHKCs) with increasing doses of LPA, although LPA does not affect cell proliferation (Supplementary Fig. 2a), mRNA expression of psoriasis-associated inflammatory factors is up-regulated after LPA treatment (Supplementary Fig. 2b). Most importantly, LPA topical treatment aggravates IMQ-induced psoriasis-like inflammation including the increasing epidermal thickness of the ear and PASI scores (Fig. 1a and Supplementary Fig. 2c), as well as Th17 and Th1 accumulation in IMQ-mediated psoriatic skin lesions (Fig. 1b). Moreover, LPA topical treatment raised transcriptional level of psoriasis-associated inflammatory factors in mice skin lesions (Supplementary Fig. 2d), indicating that LPA facilitates IMQ-induced psoriasis-like inflammation. However, Th17 and Th1 differentiation is not significantly affected by LPA (Supplementary Fig. 3), indicating that LPA regulates psoriasis pathogenesis through KCs.

**Fig. 1:**
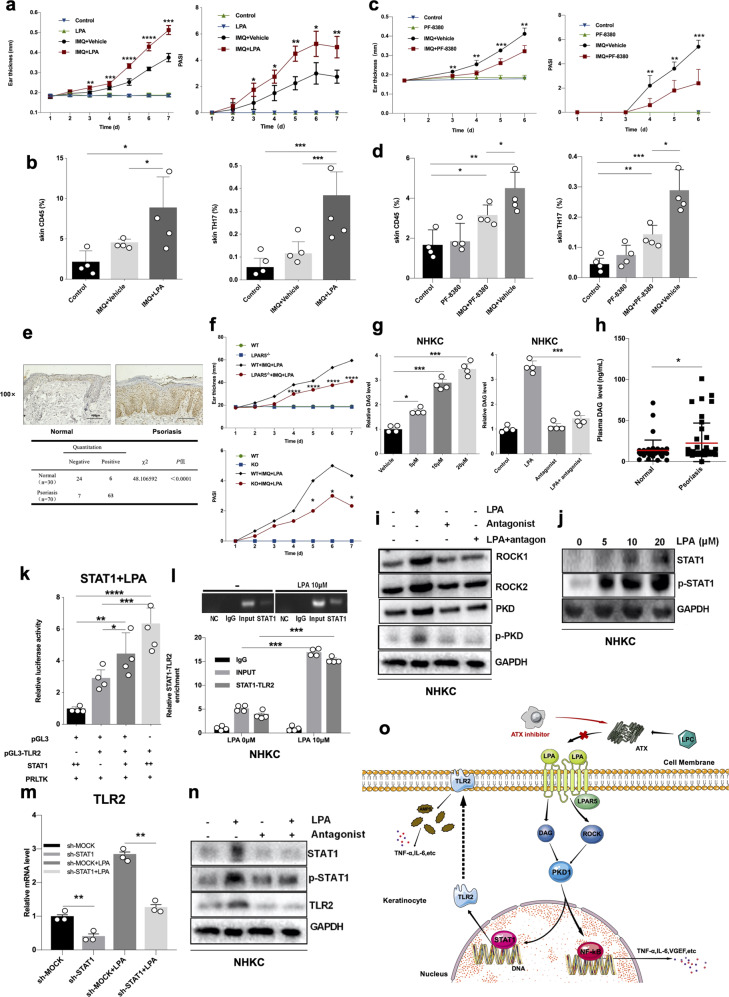
LPA promotes the pathogenesis of psoriasis by activating the inflammatory function of keratinocytes. **a** The ear thickness (Left panel) and PASI (Right panel) scores were used to assess skin lesions in mice after IMQ or IMQ plus LPA treatment. Significant differences were evaluated using a two-way ANOVA, *n = 6, *p* < 0.05. **b** Flow cytometric analysis of CD45^+^ and Th17 cells in ear lesion from mice after IMQ or IMQ plus LPA treatment, significant differences were evaluated using a two-way ANOVA, *n = 4, *p* < 0.05. **c** The ear thickness (Left panel) and PASI (Right panel) scores were used to assess skin lesions in mice after IMQ or IMQ plus ATX inhibitor treatment. Significant differences were evaluated using a two-way ANOVA, *n = 6, *p* < 0.05. **d** Flow cytometric analysis of CD45^+^ and Th17 cells in ear lesion from in above experiment, significant differences were evaluated using a two-way ANOVA, *n = 4, *p* < 0.05. **e** Representative image of the IHC analysis of LPAR5 expression in psoriatic skin lesion (*n* = 70) and healthy control (*n* = 30) (Upper panel). Statistical analysis of LPAR5 expression via IHC, Scale bars=100 μm (Lower panel). **f** The ear thickness (Upper panel) and PASI (Lower panel) scores were used to assess skin lesions in wild-type and LPAR5^-/-^ mice after IMQ or IMQ plus LPA treatment. Significant differences were evaluated using a two-way ANOVA, *n = 6, *p* < 0.05. **g** Normal human keratinocytes isolated from foreskin as described in Materials and Methods were treated with different dosage LPA as indicated for 60 min (Left panel) and treated with different dosage LPA or LPA plus LPAR5 inhibitor as indicated (Right panel), culture supernatants were collected, and ELISA analysis was performed to determine the DAG production as described in Materials and Methods. Data from multiple experiments are expressed as the means ± S.D. Significant differences were evaluated using two-way ANOVA, *p* < 0.05. **h** ELISA analysis was performed to determine the DAG production in blood of psoriasis patients (*n* = 42) and healthy controls (*n* = 42). *p*-Values were determined using one-way ANOVA; *p < 0.01; **p < 0.001; ***p < 0.0001. **i** Normal human keratinocytes were treated with LPA for 60 min, following pre-treated with LPAR5 inhibitor or vehicle for 2 h, western-blotting was performed to detect protein expression as indicated. **j** NHKCs were treated with different dosage LPA for 60 min, western-blotting was performed as indicated. **k** The TLR2 luciferase reporter gene was generated as described in Materials and Methods. PGL3-TLR2 and Stat1 were transfected into 293 T cells and double luciferase reporter gene assay was performed as described in Materials and Methods. Data from multiple experiments are expressed as the means ± S.D. Significant differences were evaluated using two-way ANOVA, *n = 4, *p* < 0.05. **l** NHKCs were treated with LPA for 60 min and ChIP assay enriched protein/DNA complexes in NHKC cells using STAT1-specific antibodies as described in Materials and Methods. Verification of the relative abundance of TLR2 gene promoter sequences in chromatin immunoprecipitation using RT-PCR with specific primers for the TLR2 promoter region. Data from multiple experiments are expressed as the means ± S.D. Significant differences were evaluated using two-way ANOVA, *n = 4, *p* < 0.05. **m** Knock down of Stat1 expression in NHKC cells were generated as described in Materials and Methods. The RNA was isolated from cells with knocking down Stat1, following in presence of LPA or not, and qRT-PCR analysis was performed to determine the mRNA expression of TLR2 in NHKC cells. Data from multiple experiments are expressed as the means ± S.D. Significant differences were evaluated using two-way ANOVA, *p* < 0.05. **n** NHKC cells were treated with LPA or LPAR5 inhibitor, western-blotting was performed as indicated. **o** After LPA signal stimulation, LPAR5 on the keratinocyte membrane activates PKD1 through ROCK and DAG, which leads to facilitate NF-κB activation and benefit expression of psoriasis-associated inflammatory factors. Meanwhile, activated PKD1 further activates Stat1, which recognizes the TLR2 gene promoter to induce its expression. TLR2 acts to be pathogen recognition receptor, which aggravates the inflammatory response in psoriasis pathogenesis

It was well-known that hydrolysis of lysophosphatidylcholine(LPC) is the most important source to produce LPA.^3^ LPC is an abundant lysophospholipid in blood and tissue, as the substrate of ATX, hydrolyzes LPC to LPA, by catalysis. ATX is a member of the extracellular nucleotide pyrophosphatase-phosphodiesterase (ENPP) protein family, which has been secreted to plasma with characteristics of lysase phospholipase PLD.^4^ To further validate the role of LPA on psoriasis, PF-8380, an ATX inhibitor, was performed to examine the effect of ATX inhibitor on psoriatic phenotype in mice. Our findings showed that compared with vehicle treatment group, PF-8380 significantly abrogated IMQ induced psoriasis-like inflammation, including reduction ear thickness and Psoriasis area and severity index (PASI) (Fig. 1c and Supplementary Fig. 4). The CD45^+^ and Th17 cell in skin infiltration were substantially suppressed in mice treated with PF-8380(Fig. 1d), suggesting that inhibition of the production of LPA could also attenuate IMQ induced psoriatic inflammation.

LPA plays a biological role through its receptors. LPA receptors are G protein-coupled receptors that can be coupled to at least one or more of the four Gα proteins, including G12/13, Gq/11, Gi/o, and Gs. Through a mining public database of psoriasis-related transcriptomes, we found that LPAR3 and LPAR5 were highly expressed in psoriasis group (Supplementary Fig. 5). Then, we validated that LPAR5 expression was significantly increased in the epidermal layer of psoriatic lesions compared to healthy control (Fig. 1e). To further study the effect of LPAR5 on psoriasis, LPAR5 knock out mouse were generated (Supplementary Fig. 6a and b) and deletion of LPAR5 expression significantly reduced the IMQ plus LPA-mediated psoriasis-like phenotype, including ear thickness and PASI scores (Supplementary Fig. 7a and Fig. 1f). Moreover, expression of psoriasis-associated inflammatory factors was dramatically down-regulated in LPAR5^-/-^mice in the presence of IMQ plus LPA (Supplementary Fig. 7b). However, deletion of LPAR5 expression in CD4 + T cells did not affect IMQ plus LPA-mediated psoriasis-like phenotype (Supplementary Fig. 6c, d), suggesting that LPAR5 plays a critical role in LPA-mediated pathogenesis of psoriasis independent with CD4^+^T cells. LPAR5 was identified through unbiased screening approaches and is coupled to G12/13 and Gq/11, initiating downstream cascades though DAG or Rock and protein kinase D1 (PKD1), respectively.^5^ Given that DAG is a key mediator of the PKD1 induced pathway, we examined DAG production in NHKC cells after LPA treatment. The results indicated that DAG production was increased in response to LPA with a dose-dependent manner, while LPAR5 antagonist attenuated LPA-induced DAG production elevation (Fig. 1g). Interestingly, we found that DAG production was raised in plasma of psoriasis patients (Fig. 1h). In addition, we found that LPA treatment dramatically increased ROCK1, ROCK2, and p-PKD1 expression in NHKC cells (Supplementary Fig. 9a), as expected, LPAR5 antagonist remarkable blocked LPA triggering downstream pathways, such as ROCK1, ROCK2, DAG and p-PKD (Fig. 1i).

Protein kinase D is a family of stress-reactive serine/threonine kinases, which is triggered by an effector of diacylglycerol (DAG) and PKC. Protein kinase D could be activated by a variety of stimulators, including growth factors, neuropeptides, hormones, regulating a variety of biological and pathological processes.^5^ Evidences showed that PKD1 phosphorylation activates IKKα/β phosphorylation, resulting in IκB degradation, followed by p65 and p50 translocation into the nucleus, and finally activates the transcriptional activity of NF-κB, which plays key roles in psoriasis pathogenesis, particular in activation of KCs.^5^ In our study, the results also exhibited that p65 and p50 were expressed in KCs in the nucleus, as well as the increased expression of p65 and p50 proteins in psoriasis induced by LPA (Supplementary Fig. 9b). Moreover, the LPA-mediated induction of the expression of psoriasis-associated inflammatory factors was suppressed by the administration of a LPAR5 antagonist (Supplementary Fig. 9c).

TLR2 belongs to one of pathogen recognition receptors (PRRs), which recognize PAMPs to initiate innate immunity or aggravate the adaptive immune response against pathogens. The associated of PAMPs with TLR2 heterodimer leads to the interaction of Type 1 IL-1 Receptor (TIR) with TIRAP, which recruits MyD88, IRAKs to form complex. The subsequent of phosphorylation IRAKs results in activation of TRAF6 to trigger NF-κB translocation into the nucleus, which raises diversity target molecules expression including pro-inflammatory cytokines.

We identified and validated that TLR2 and STAT1 are key molecules regulated by LPA using RNA-Seq in KCs (Supplementary Fig. 10). STAT1 is a member of the STAT family, which involved in the activation of multiple signaling pathways. STAT1 is significantly increased in psoriasis skin lesion. Our finding showed that LPA dramatically upregulates p-Stat1 and non-Stat1 expression (Fig. 1j). Interestingly, STAT1 recognized its DNA motif and bound to the TLR2 promoter as evidenced by luciferase and CHIP assays (Fig. 1k, l and Supplementary Fig. 10a, b), whereas knock-down of STAT1 abrogated LPA-induced psoriasis-related TLR2 expression (Fig. 1m and Supplementary Fig. 10c). Moreover, LPAR5 inhibitor dramatically attenuated LPA-mediated increases in STAT1, p-STAT1 and TLR2 expression in KCs (Fig. 1n), indicating LPA activates keratinocytes via a mechanism that is at least partially dependent on the STAT1-TLR2 axis.

In summary, our study elucidates the role of the LPA/LPAR5 axis in keratinocytes in the pathogenesis of psoriasis (Fig. 1o), contributing to our understanding of the pathogenesis of psoriasis. Furthermore, our results also demonstrate that the LPA/LPAR5 axis is a potential and promising target in psoriasis therapy.

## Supplementary information

Supplementary Materials for Lysophosphatidic acid mediates the pathogenesis of psoriasis by activating keratinocytes through LPAR5

## Data Availability

Data that support the findings of this study have been deposited in NCBI with the BioProject accession number “PRJNA650250”.
