# Supplementary material for: Lysophosphatidic acid mediates the pathogenesis of psoriasis by activating keratinocytes through LPAR5
Source: Signal Transduct Target Ther. 2021 Jan 15;6:19. doi: 10.1038/s41392-020-00379-1 (PMC7810744; doi:10.1038/s41392-020-00379-1)
Supplement: Supplementary file 1 — Supplementary Materials for Lysophosphatidic acid mediates the pathogenesis of psoriasis by activating keratinocytes through LPAR5 [file 41392_2020_379_MOESM1_ESM.docx]

**Supplementary Materials for**

Lysophosphatidic acid mediates the pathogenesis of psoriasis by activating keratinocytes through LPAR5

Li Lei^1,2,3,4,5†^ , Bei Yan^1,2,3,4,5†^ ，Panpan Liu^1,2,3,4,5^, Jie Li^1,2,3,4,5^, Chao Chen^1,2,3,4,5^, Wu Zhu^1,2,3,4,5^, Yehong Kuang^1,2,3,4,5^,Xiang Chen^1,2,3,4,5*^, Cong Peng^1,2,3,4,5*^

^1^The Department of Dermatology, Xiangya Hospital, Central South University

^2^National Clinical Research Center for Geriatric Disorders，Xiangya Hospital

^3^Hunan Key Laboratory of Skin Cancer and Psoriasis，Xiangya Hospital

^4^Hunan Engineering Research Center of Skin Health and Disease, Xiangya Hospital

^5^Xiangya Clinical Research Center for Cancer Immunotherapy, Central South University

†These authors contributed equally to this work.

^*^Correspondence: Cong Peng([pengcongxy@csu.edu.cn](mailto:pengcongxy@csu.edu.cn)),

Xiang Chen([chenxiangck@126.com](mailto:chenxiangck@126.com))

**This PDF file includes:**

Materials and Methods

Supplementary Text

Figures. S1 to S10

Tables S1 to S4

**Materials and methods**

Skin and blood samples of Psoriasis and Mice

The skin and blood samples of patients in this study were donated from psoriasis patients in the dermatology clinic of Xiangya Hospital of Central South University. The patient was diagnosed as psoriasis vulgaris by an experienced dermatologist according to typical clinical manifestations combined with histopathological examination. The normal control sample came from the healthy volunteers in the physical examination center or the healthy volunteers recruited (sex and age matched with the patient), approved by local ethics Institutional Review Board (IRB) (Xiangya Hospital, Central South University, IRB-20180312). Mice follow the 3R principle, pay attention to the welfare of experimental animals, approved by local ethics Institutional Review Board (IRB) (Xiangya Hospital, Central South University, IRB-20180311).

Antibodies, plasmids, and drugs

Primary antibodies against STAT1 (Abcam, ab31369), p-STAT1 (Abcam, ab29045), TLR2 (Abcam, ab213676), p65 (CST, 33593S), p50 (CST, 13586S), ROCK1 (Abcam, ab45171), ROCK2 (Abcam, ab66320), PKD (CST, 90039S), p-PKD (CST, 2054S), GAPDH (Proteintech, 10494-1-AP), β-actin (Santa Cruz, 47778), and laminAC (Santa Cruz, 20681), and goat anti-mouse secondary antibody (ABclonal, AS003) or goat anti-rabbit secondary antibody (ABclonal, AS014) for Western blotting detection. Fluorescence-labeled antibodies from BD Biosciences: FITC-conjugated anti-CD4，PE-conjugated anti-IL-17A，APC-conjugated anti-IFN-γ, APC-CY7–conjugated anti-CD45.Cells were cultured in DMEM or RPMI 1640 medium supplemented with 10% fetal bovine serum (Biological Industries, Israel), penicillin (Gibco), streptomycin (Gibco), glutamine (Gibco) and sodium pyruvate (Gibco). LPA (Avanti, 857128P), LPAR5 antagonist (APE x BIO, B5689), shSTAT1(GeneChem,6772) used to stimulate cells. Antibody for immunohistochemical staining: anti-LPAR5 (Abcam, ab140837). Drugs for the treatment of psoriatic dermatitis-like mouse model: IMQ cream (5%) (Sichuan Mingxin Pharmaceutical Co., Ltd., #120503), LPA (Avanti, 857128P), PF-8380 (Selleck，S8218).

Cell culture experiments

293T cells were purchased from ATCC and maintained in DMEM (Biological Industries, Israel) supplemented with 10% FBS(Biological Industries, Israel) at 37 °C and 5% CO2. Human primary keratinocytes were extracted from foreskin tissue donated by circumcision patients (informed consent was obtained). Mice primary keratinocytes were extracted from the skin of newborn C57BL/6 mice (approved by the Ethics Committee). The specimens were soaked in DPBS(BI) containing triple antibodies for 2 hours, cut off the connective tissue and put into 2mg/ml dispase（Sigma）to digest overnight. The dermis and epidermis were separated the next day. The epidermis was cut and digested in trypsin containing EDTA0.25%(Gibco) for 10 minutes at 37 °C and 5% CO2. Then the same amount of DMEM containing 10%FBS was added to terminate the digestion. Finally, the primary keratinocytes were maintained in keratinocyte growth medium 2 (C20011, PromoCell). Cells were cultured in RPMI 1640 medium supplemented with 10% FBS(Biological Industries, Israel), penicillin (Gibco), streptomycin (Gibco), glutamine (Gibco) and sodium pyruvate (Gibco).

Psoriasis-like lesion mice model

IMQ-induced psoriasis-like skin inflammation was induced in 6- to 8-week-old mice (C57BL/6 background) by a daily topical dose of 62.5 mg of IMQ cream (5%) (Sichuan Mingxin Pharmaceutical Co., Ltd., #120503) on the shaved back skin for six consecutive days. For ear skin IMQ application model, 6- to 8-week-old mice (C57BL/6 background) were treated with 15.625 mg of IMQ cream on the ear for six days. The ear thickness was measured daily. For LPA treatment, the ear skin was subcutaneously injected with 0.25 mM LPA (Avanti, 857128P soluble in normal saline) or normal saline once a day during the application of IMQ in mice for one week. For PF8380 treatment, the intragastric administration with 30mg/kg PF-8380 (Selleck，S8218 soluble in normal saline) or normal saline twice a day during the application of IMQ in mice for one week. In order to evaluate the severity of skin inflammation in mice, an objective scoring system based on the clinical area and severity index of psoriasis (PASI) was used. The severity of erythema and scales were 0,none;1,mild;2, moderate;3,marked;4,severe. Measure the ear thickness with a caliper. Daily information on food intake, body weight, PASI score, and ear thickness were recorded.

MTS assay

Keratinocytes were stimulated with different concentrations of LPA for 24 h, 48 h and 72 h, and then MTS/PMS was added (20:1) (Promega) mixture was incubated at 37 ℃ for 2 h, and then the absorbance was determined by 490nm wavelength of enzyme labeling instrument. The absorbance value was ordinate. GraphPad Prism pad software was used to draw cell growth curve and SPSS software was used for statistical analysis.

Plasmid transfection and virus transduction

For STAT1 silencing, the lentiviral particles expressing shRNAs targeting STAT1 and one scramble control shRNA were purchased from GeneChem, Shanghai, China. For transfecting STAT1 silencing plasmid into HEK293T, cells were seeded at density of 6 × 10^6^ cells per 100 mm dish. For transducing STAT1 silencing lentivirus into NHKCs, silence of Stat1 in NHKCs, cells were seeded into 24-well plate at 1 × 10^4^ cells/well with DMEM and 10% FBS. After 24 hours, NHKC cells were infected in the medium supplemented with 10 μ g / mL polyallene by changing the fresh culture medium. Stable transduced cells were selected with 2 μg/mL puromycin after 48 h transfection. The transfection efficiency was verified by western blotting (Supplementary Figure 7).

Quantitative real-time PCR

RNA from tissues or cells was extracted from cells using Trizol (Thermo Scientific). After quantitative analysis of RNA using a nucleic acid concentration analyzer (NanoDrop, Thermo Scientific), cDNAs were generated by reverse transcriptase using HiScript Q RT SuperMix (Thermo Scientific, #K1622). Real-time quantitative PCR was performed to assess the expression of targeting molecules . Assessment of mRNA expression was performed using SYBR Green PCR Master Mix (ComWin Biotech, Beijing). β-Actin or GAPDH was used as an internal control. The standard 2^-△△Ct^ method was used to determine the relative gene expression to internal control. The primer sequences of above the genes are presented in Supplementary material table 1.

HE and immunohistochemical staining

Skin tissues embedded in paraffin were cut into 5-mm slides. After the sections were deparaffinized, they were stained with hematoxylin staining solution and eosin staining solution. After dehydration with a gradient of different concentrations of ethanol, the sections were sealed using a neutral quick-drying gel-sealed tablet (GENWIN Biotech, Guangzhou). Then, the HE staining results were observed under a microscope. After dehydration, the antigen in the sections was repaired by sodium citrate/EDTA antigen repair solution (Google Biotech, Wuhan). Then, the sections were blocked with 1% bovine serum albumin. Sections were incubated with anti-LPAR5 (Abcam, ab140837) at 4℃overnight. After adding primary and secondary anti-reinforcing agents (Zsbio, Beijing), the sections were treated with biotinylated goat anti-rabbit antibody (Proteintech) for 45 minutes. After sections were treated by DAB, the nuclei were stained with hematoxylin. After dehydration and sealing, the immunostaining result was observed under a microscope. Experimental results were evaluated by two investigators. Cell proportions were scored as follows: 0 (<5% positive cells); 1 (5%–25% positive cells); 2 (26%–50% positive cells); 3 (51%–75% positive cells); and 4 (>76% positive cells). Staining intensity was graded as follows: 0 (colorless), 1 (light yellow), 2 (yellow brown), and 3 (brown). The above two scores were multiplied to obtain the total score. A score of 3 or greater is judged as positive.

High-throughput Sequencing

Transcriptomic changes after the subcutaneous injection of 0.25 mM LPA daily for one week in the ear skin of the psoriasis-like lesion mice model were analyzed using the BGISEQ-50 high-throughput sequencing platform (BGI, Shenzhen, China).

Western blotting

Tissues and cells were lysed with RIPA lysis buffer (Beyotime) containing PMSF and phosphorylated protease inhibitors. In addition, nuclear and cytoplasmic proteins were extracted using nuclear and cytoplasmic extraction reagents (Thermo Scientific, 78835) according to the operating instructions. Briefly, 30 to 40 μg protein per well were separated by 10%-12% SDS-polyacrylamide gels. Then, proteins were transferred to PVDF membranes. After blocking with sealing liquid, the membranes were incubated with primary antibodies against STAT1 (Abcam, ab31369), p-STAT1 (Abcam, ab29045), TLR2 (Abcam, ab213676), p65 (CST, 33593S), p50 (CST, 13586S), ROCK1 (Abcam, ab45171), ROCK2 (Abcam, ab66320), PKD (CST, 90039S), p-PKD (CST, 2054S), GAPDH (Proteintech, 10494-1-AP), β-actin (Santa Cruz, 47778), and laminAC (Santa Cruz, 20681) overnight at 4 °C. After PBST washes, goat anti-mouse secondary antibody (ABclonal, AS003) or goat anti-rabbit secondary antibody (ABclonal, AS014) was used to incubate membranes for 30–60 mins. Protein levels were evaluated after ECL visualization.

Flow cytometry

After being stimulated by LPA, the keratinocyte suspension was prepared in PBS. Detection was then performed on a FACSCalibur flow cytometer (BD Biosciences) using Cell-Quest software (BD Biosciences).

For obtaining single-cell suspensions, mouse ear skin was incubated in dispase (5 mg/ml) to separate epidermis from dermis. In order to obtain cell suspension, mouse ear skin was incubated with dispase (5 mg/ml) at 37°C for 3 hours to separate epidermis and dermis. Collagenase IV and DNase I were used to obtain dermal suspensions. With the flow cytometry using CytoFlex (Beckman Coulter) and the following fluorescence-labeled antibodies from BD Biosciences: FITC-conjugated anti-CD4，PE-conjugated anti-IL-17A，APC-conjugated anti-IFN-γ, APC-CY7–conjugated anti-CD45. In order to detect Th1 and Th17, 100 ng/ml stimulator was needed to stimulate for 5 h in vitro. After the cell surface staining, the cells were fixed and permeable, and the cells were stained with IL-17A and IFN-γ. Samples were harvested analyzed using FlowJo software (Tree Star).

Luciferase reporter assays

The target gene was obtained by polymerase chain reaction (PCR), and primers were designed for the target site. And the appropriate enzyme digestion buffer was selected according to the restriction site. PGL3 is a luciferase reporter vector (Promega, E1751). PRLTK is a renilla luciferase control report vector (Promega, P100001). HEK293T cells were maintained in DMEM supplemented with 10%FBS, glutamine, penicillin and streptomycin. 2.0× 10^4^ 293T cells were seeded in the wells of a 24-well plate 1 day before transfection, and each well was transfected with a mixture of 10ul luciferase reporter vector pGL3-TLR2 plasmid, STAT1 plasmid, or pRLTK internal reference plasmid (GenePharma Biotechnology Company Shanghai, China). STAT1-knockdown HaCaT and NHKC cells were transfected with pRLTK plasmids. After 24 hours of transfection, the firefly and renilla luciferase activities were both evaluated using the Dual Luciferase Reporter Assay system (PROMEGA). The relative luciferase activities were calculated by the ratio of firefly/renilla luciferase activity.

Chip assay

After collecting a sufficient number of keratinocytes treated by LPA or not, the cells were subjected to crosslinking, lysis, and DNA breakage treatment. ChIP assays were used to enrich the protein/DNA complexes in keratinocytes using STAT1-specific antibodies (CST, 14994S). The relative abundance of TLR2 gene promoter sequences in the chromatin immunoprecipitation reaction was verified by using qPCR with specific primers for the TLR2 promoter region. For qPCR quantification, the following primer pairs were used: forward, 5' CTGTCGCAGCCTAGCTCACGG 3'; reverse, 5' GCCGGAGGGAACTCTGGACC 3'.

RNA-sequencing (RNA-Seq)

After local smearing of IMQ, the ear lesions of mice in LPA or control group were put into a cryopreservation tube filled with RNA protective solution and handed over to Wuhan Huada sequencing Company (www.genomics.org.cn, BGI, Shenzhen). The BGISEQ-500 platform provided by them was used for RNA sequencing analysis.

ELISA

The serum or cell culture supernatant was collected and tested with ELISA kit (Shanghai Jianglai Biology, jL13897) according to the manufacturer's instructions, and the concentration of DAG was detected by 450nm wavelength of enzyme labeling instrument.

Generation of LPAR5-knockout mice

LPAR5 knockout heterozygous C57BL/6J mice constructed by CRISPR/Cas9 technology were provided by Shanghai Model Organisms Center, Inc. Heterozygous mice (LPAR5^+/-^) were mated with each other to obtain homozygous mice (LPAR5^-/-^) in which the LPAR5 gene was completely knocked out. P1 and P2 with P3 and P4 were used to genotype the LPAR5 deleted allele (2785bp) and (332bp).P1, 5' CCTCCCAGCACCTTTCTAAC3'.P2,5'CCACATGCCAGGTGTCAAGT3'.P3,5'ATGGTTAGGTCACTCAAGCT3'.P4, 5' CCACATGCCAGGTGTCAAGT3' .The knockout LPAR5 mice did not form any skin phenotype under basic conditions for at least 6 months of life and reproduction.

Generation of LPAR5fl/fl and LPAR5fl/flCD4Cre mice.

LPAR5fl/fl C57BL/6J mice constructed by CRISPR/Cas9 technology were provided by Shanghai Model Organisms Center, Inc. To establish loxP-LPAR5-loxP mice, a targeting vector was designed to insert a loxP site upstream of LPAR5 and a second loxP site downstream of LPAR5. Cre recombinase can recognize loxP site. If two loxP sites are introduced into the genome site in the same direction, the expression of Cre will lead to the deletion of loxP flanking DNA sequence. The heterozygote of loxP-LPAR5-loxP mice was hybridized with CD4-Cre transgenic mice with C57BL/6J background. LPAR5 allele (1870bp) and CD4 allele (168bp) were genotyped by P5 and P6 genes. P5 and P6 were used to genotype the LPAR5 floxed allele (1,870 bp) and the LPAR5fl/fl deleted allele (168 bp). P5, 5' TTCAGAGCAAATCTTCTGAGGC 3' ; P6, 5' CCATGTACCAGCTCTTAGCCAT 3' . Excision by CD4-Cre was complete for all pups used in experiments. The LPAR5fl/fl and cKO mice did not form any skin phenotype under basic conditions for at least 6 months of life and reproduction.

Statistical analysis

Statistical analysis was performed with Prism 8 (GraphPad Software) or SPSS 22.0. Data are presented as the mean±SD. All experiments were repeated in triplicate at a minimum. ANOVA followed by a comparison between two groups using Student’s t-test was used throughout this study. P< 0.05 was considered statistically significant. (*P < 0.05, **P < 0.01, ***P < 0.001, ****P < 0.0001).

Supplemental Text

Acknowledgments

This work was supported by National Natural Science Foundation of China Grant No. 81773341,81830096, 81430075, 81673065, 81620108024.

Conflict of interest
The authors declare that they have no conflict of interest.

Author contributions

LL and BY performed most of the experiments, analyzed the data. CC assisted with luciferase assays. PL assisted with ChIP experiments. WZ and YK collected the clinical samples. JL provided technical support and suggestions for the project. XC and CP conceived the project and supervised the study. LL，BY and CP wrote the manuscript.

Data availability statement

Data that support the findings of this study have been deposited in NCBI with the BioProject accession number “PRJNA650250”.


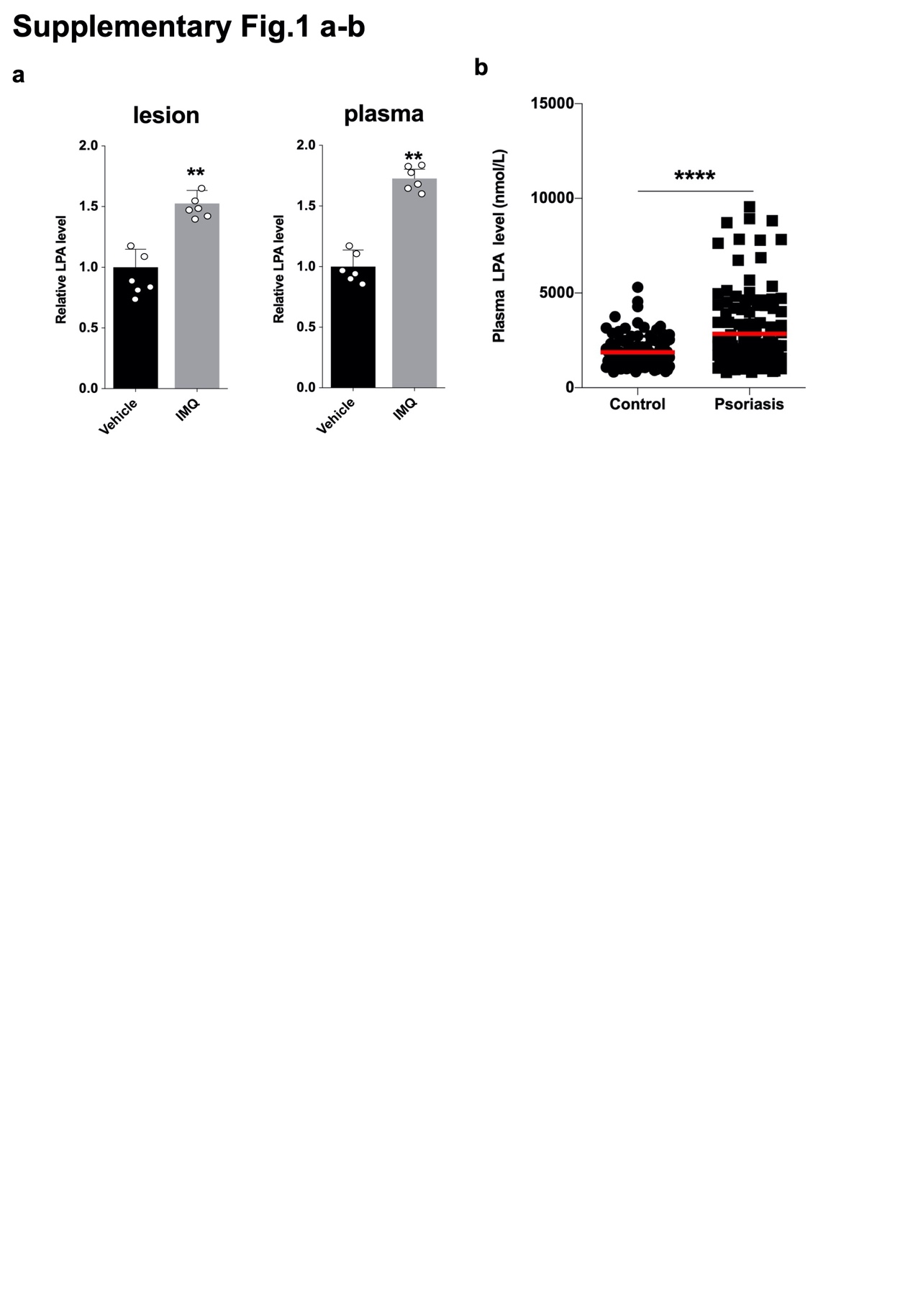
Figure. S1.

**Figure S1.LPA is elevated in psoriasis patients and IMQ induced mice .** (a) ELISA analysis was performed to determine the LPA level in the skin lesions and plasma of model and control mice as described in *Materials and Methods*. The LPA level in the skin lesions and plasma of model and control mice was measured. (b) ELISA analysis was performed to determine the LPA level in psoriasis patients (n=120) and healthy volunteers (n=100). Data are representative of three independent experiments. Data are presented as the mean±SD. P-values were determined using one-way ANOVA; *, P < 0.01; **, P < 0.001; ***, P < 0.0001.

Figure.S2.

**
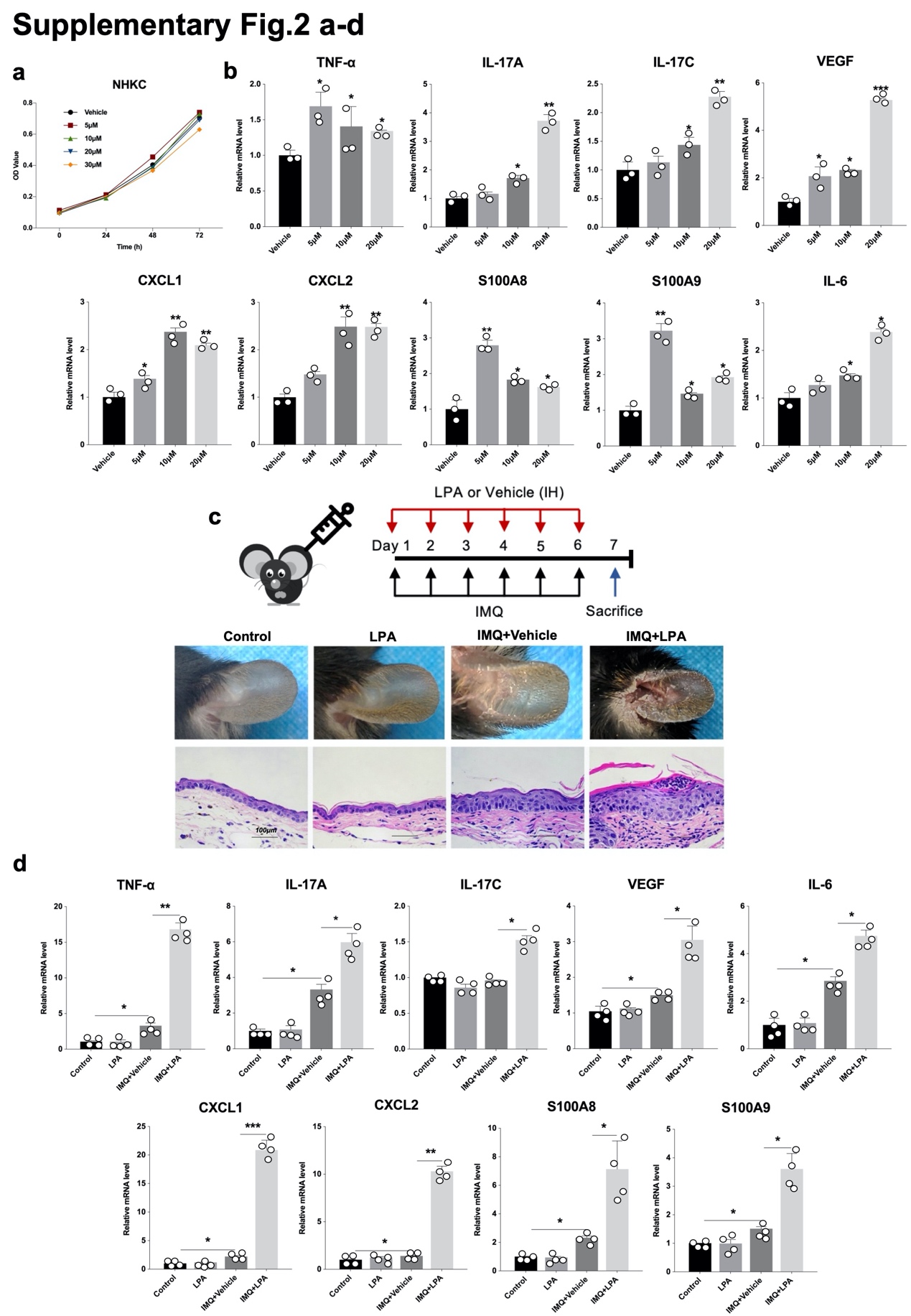
**

**Figure.S2.LPA promotes the pathogenesis of psoriasis keratinocytes.** (a) Normal human keratinocytes were isolated from foreskin as described in *Materials and Methods* and treated with different dosage LPA as indicated. The cell viability was detected by MTS as described in *Materials and Methods*. Data from multiple experiments are expressed as the means ± S.D. Significant differences were evaluated using two-way ANOVA, and the asterisk (*) indicates a significant difference (P < 0.05). (b) Normal human keratinocytes isolated from foreskin as described in *Materials and Methods* were treated with different dosage LPA as indicated for 12hrs. RNA was isolated and qRT-PCR analysis was performed to determine the mRNA expression of psoriasis-associated inflammatory factors as indicated. Significant differences were evaluated using a two-way ANOVA, p<0.05. (c) Schematic diagram of IMQ plus LPA or vehicle were applied topically to mice daily(Upper panel). Macroscopic phenotypical representations of psoriasis-like ear skin lesions in mice (one representative mouse is presented, n=6) and H&E staining showed ear skin after IMQ or IMQ plus LPA treatment (original magnification 100x),Scale bars=100μm(lower panel). (d) RNA was isolated from ear skin of mice treated with IMQ or IMQ plus LPA and qRT-PCR analysis was performed to determine indicated mRNA expression（n=4）.Data are representative of three independent experiments .Data are presented as the mean±SD. P-values were determined using two-way ANOVA; *, P < 0.01; **, P < 0.001; ***, P < 0.0001.

Figure. S3.

**
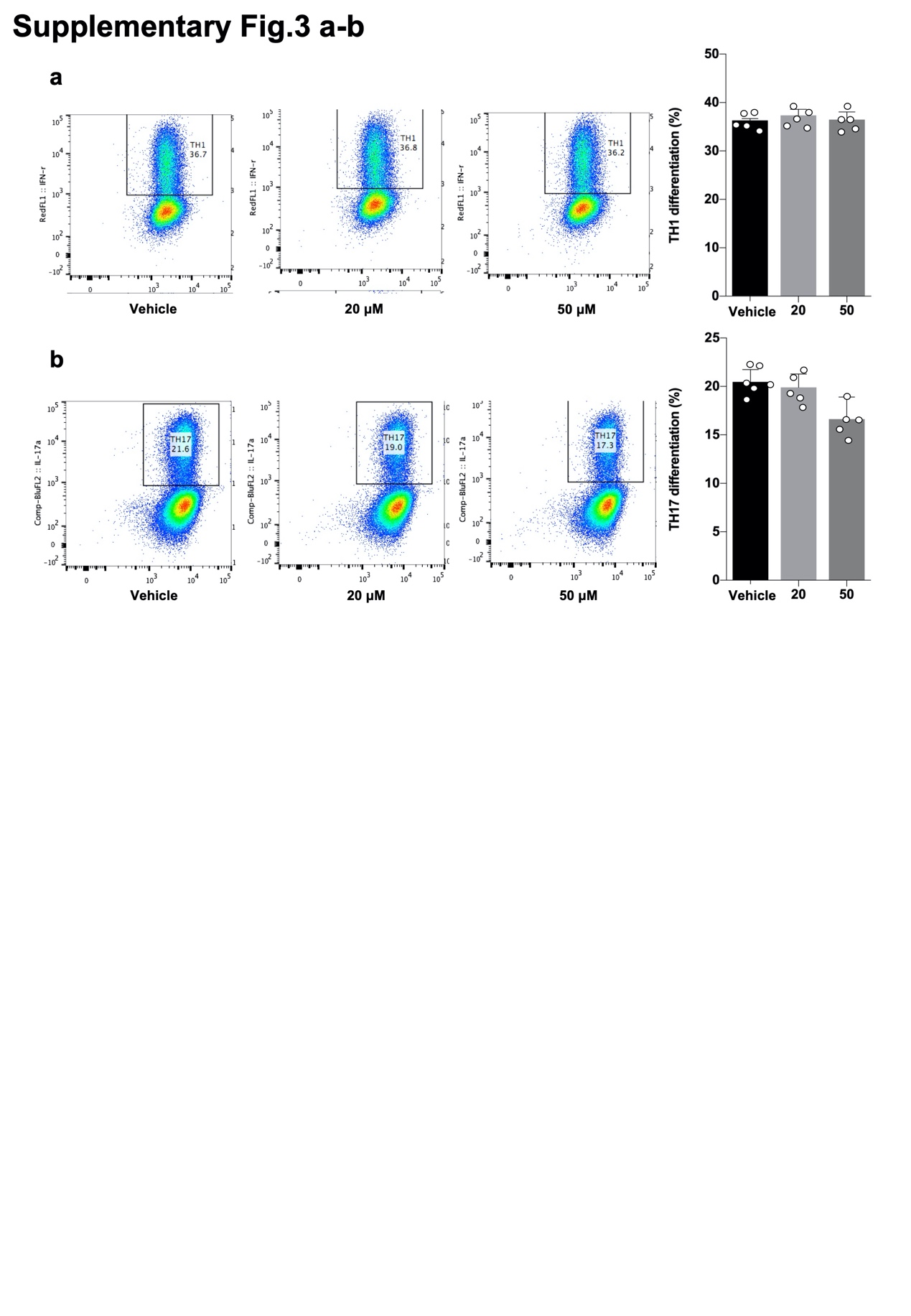
**

**Figure. S3.LPA does not affect Th17 and Th1 differentiation.** Medium contained agents to induce of T cell subsets. (a-b) Flow cytometric analysis of Th17 and Th1 differentiation in mouse naïve T cells were treated with different dosage LPA as described in *Materials and Methods* (n=5). Data are representative of three independent experiments. P-values were determined using one-way ANOVA; *, P < 0.01; **, P < 0.001; ***, P < 0.0001.

Figure.S4.

**
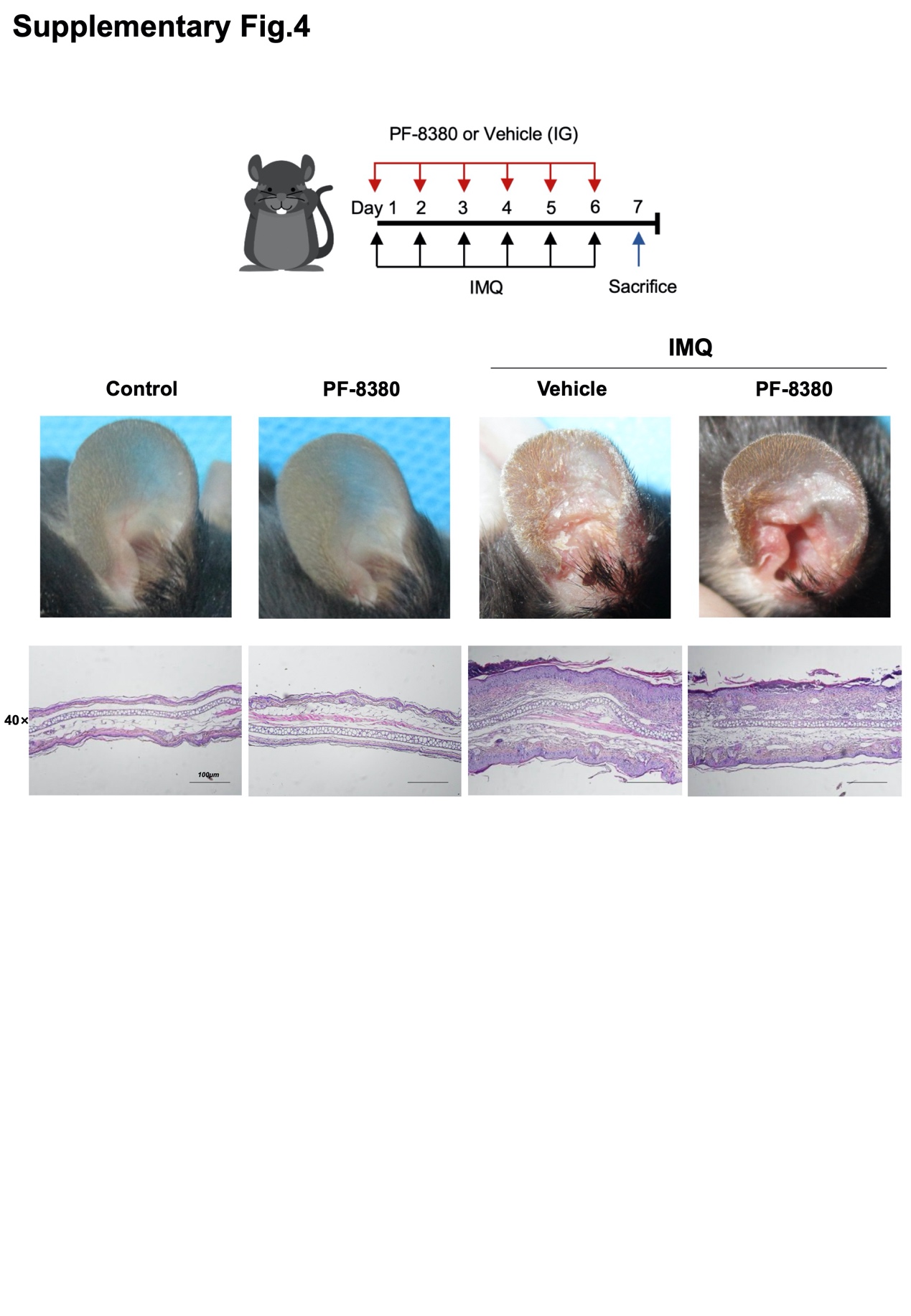
**

**Figure.S4.ATX inhibitor attenuates IMQ-induced murine psoriasiform dermatitis.** Schematic diagram of IMQ plus ATX inhibitor or vehicle were applied topically to mice daily(Upper panel). Macroscopic phenotypical representations of psoriasis-like ear skin lesions in mice (one representative mouse is presented, n=6) and H&E staining showed ear skin after IMQ or IMQ plus ATX inhibitor treatment (original magnification 100x), Scale bars=100μm(Lower panel).

Figure. S5.

**
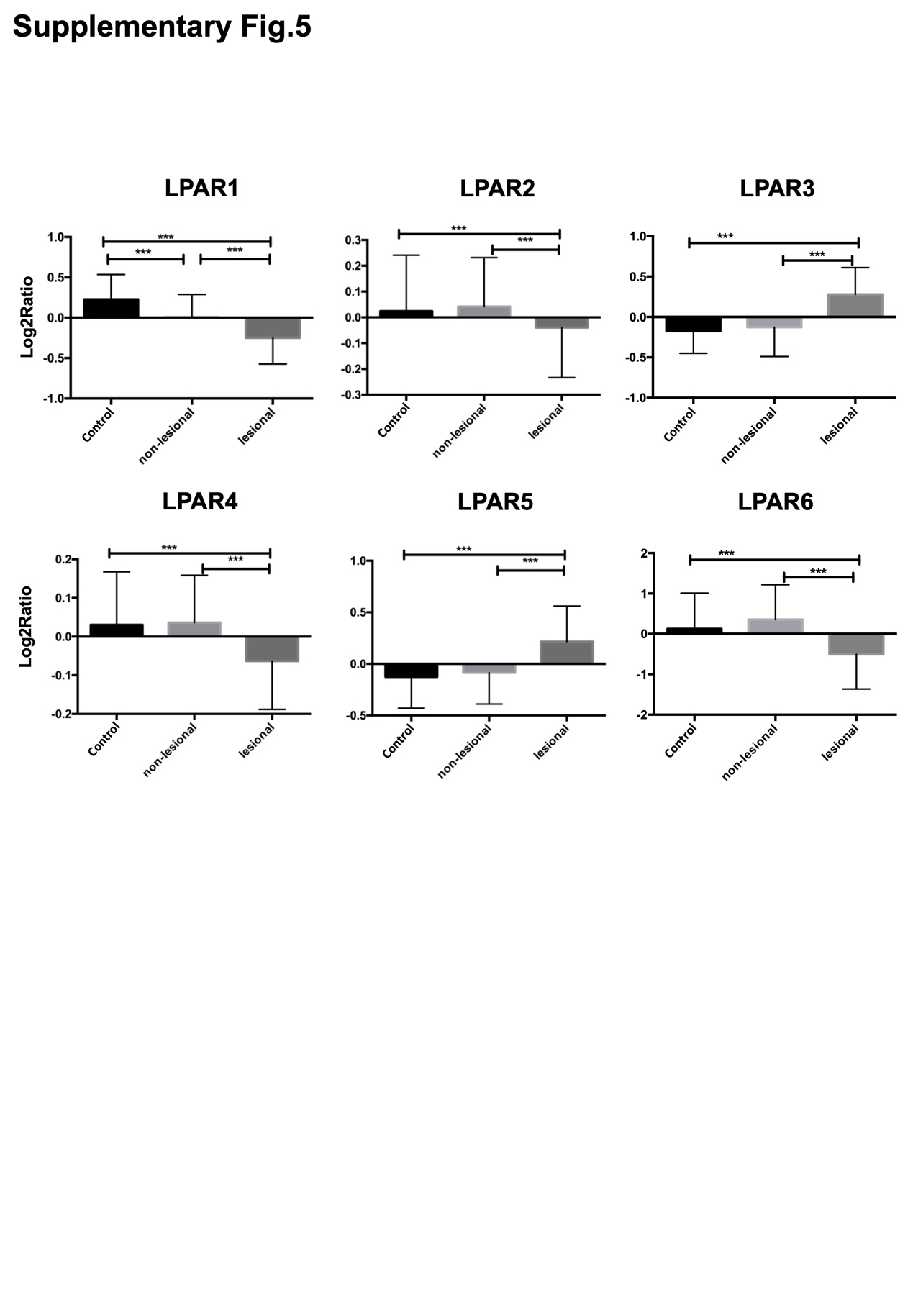
**

**Figure.S5.LPAR5 was over-expressed in psoriasis skin lesions.** Through a mining public database (GEO: 13355) of psoriasis-related transcriptomes, we found that LPAR5 was overexpressed in psoriasis skin lesions. Control (n)=64; psoriasis nonlesional skin (n)=58; psoriasis lesional skin (n)=58.

Figure. S6.

**
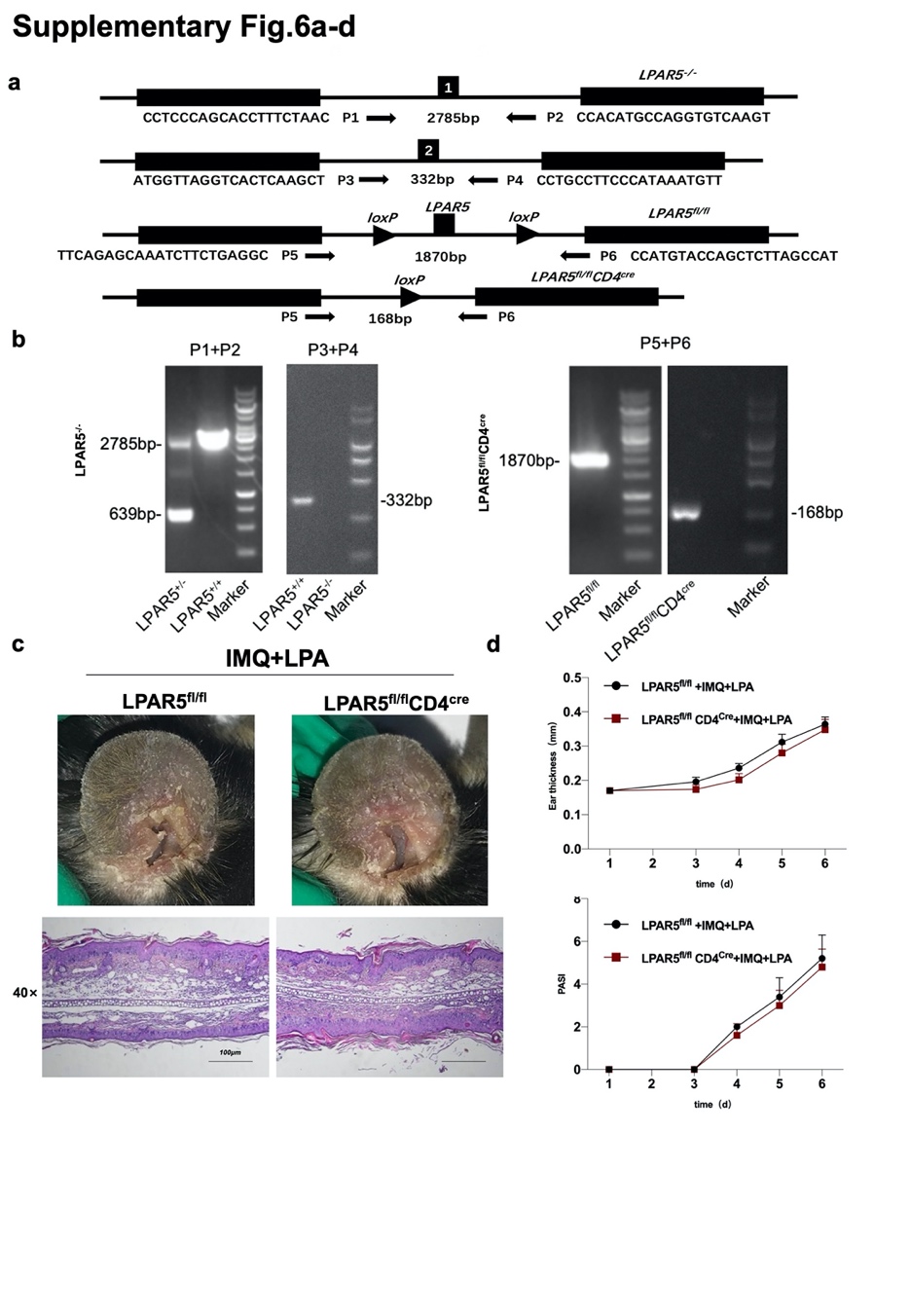
**

**Figure.S6.Investigating the role of LPAR5 in regulating psoriasis progression in LPAR5^fl/fl^/LPAR5^fl/fl^ CD4^cre^ mice.**(a) Schematic diagram of primers for genotyping and targeting strategies. (b) LPAR5^-/-^ genotyping was carried out by P1/P2 and P3/P4, 1870 bp band for LPAR5^fl/fl^ and 168 bp band for LPAR5^fl/fl^CD4^cre^ genotyping was carried out by P5/P6, DNA samples were prepared from total skin. (c) Treating LPAR5^fl/fl^ control mice and LPAR5^fl/fl^ CD4^cre^ mice with IMQ followed by subcutaneous injection of LPA or vehicle. Macroscopic phenotypical representations of psoriasis-like ear skin lesions in mice (one representative mouse is presented, n=6) and H&E staining showed ear skin after IMQ plus LPA or vehicle treatment (original magnification 100x), Scale bars=100μm(lower panel). (d) The ear thickness (Upper panel) and PASI(Lower panel)scores were used to assess skin lesions in mice after IMQ plus LPA or vehicle treatment(n=6). Significant differences were evaluated using a two-way ANOVA, *, P < 0.01; **, P < 0.001; ***, P < 0.0001.

Figure.S7.

**
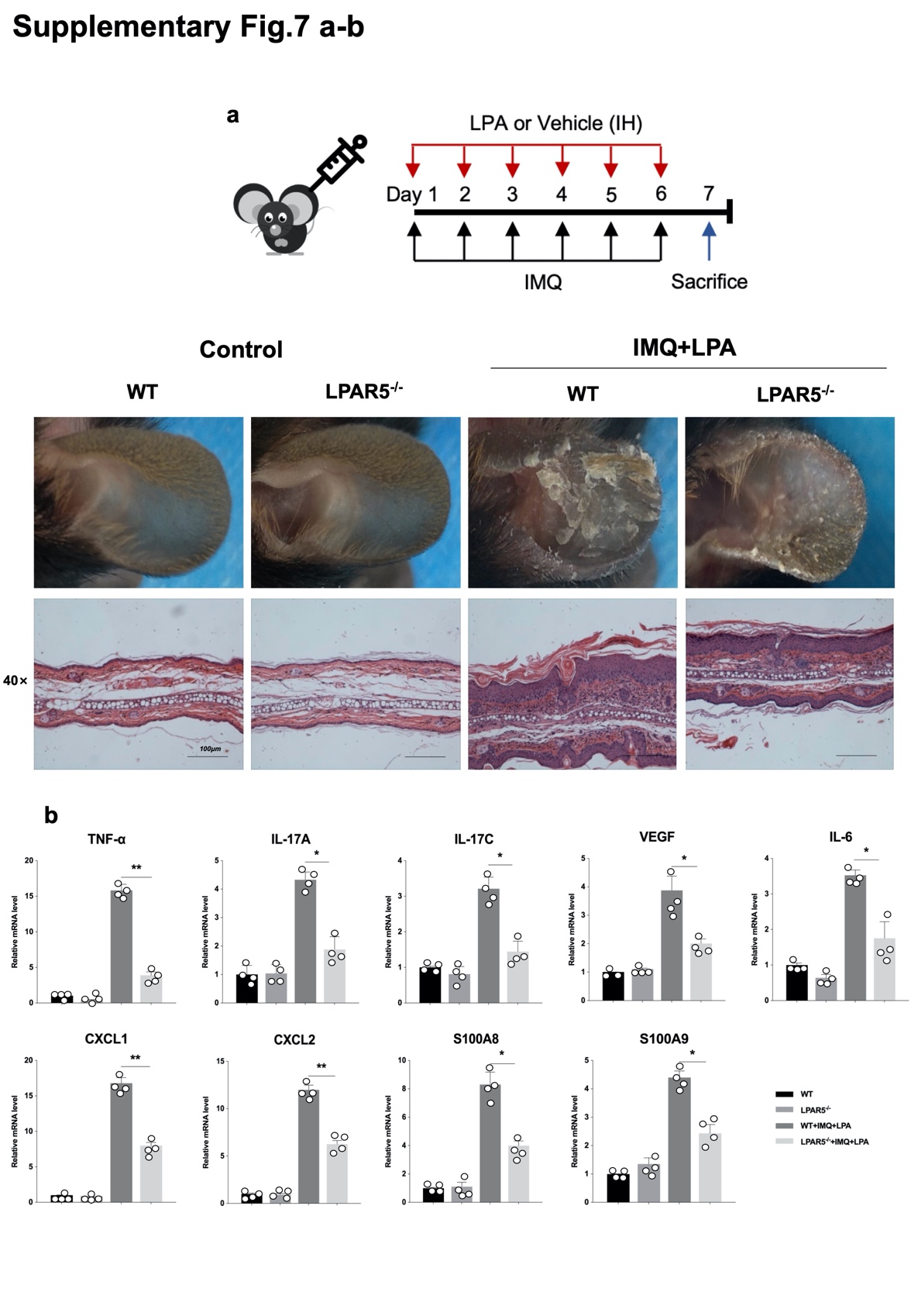
**

**Figure.S7.Study the role of LPAR5 in psoriasis pathogenesis through LPAR5-knockout mice.** (a) Schematic diagram of IMQ plus LPA or vehicle were applied topically to ear skin of wild-type and LPAR5^-/-^ mice daily(Upper panel). Macroscopic phenotypical representations of psoriasis-like ear skin lesions in mice (one representative mouse is presented, n=6) and H&E staining showed ear skin after IMQ or IMQ plus LPA treatment (original magnification 100x), Scale bars=100μm(lower panel). (b) RNA was isolated from ear skin of mice treated with IMQ or IMQ plus LPA and qRT-PCR analysis was performed to determine indicated mRNA expression(n=4). Data are representative of three independent experiments. Data are presented as the mean±SD. P-values were determined using two-way ANOVA; *, P < 0.01; **, P < 0.001; ***, P < 0.0001.

Figure.S8.

**
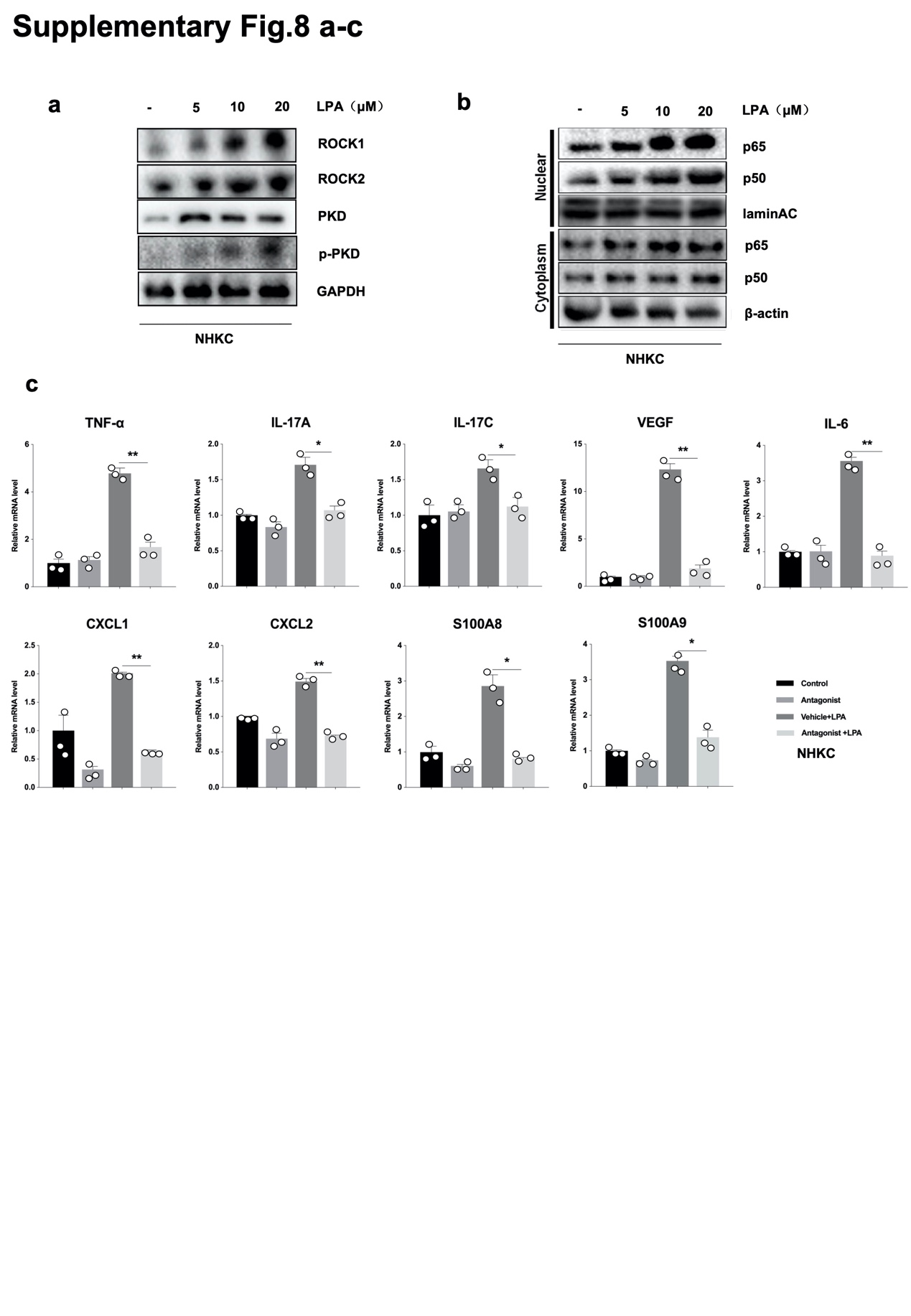
**

**Figure.S8.LPA activates the Rho/DAG-PKD signaling pathway through LPAR5.** (a) Normal human keratinocytes were treated with different dosage LPA as indicated for 60min, the protein level of ROCK1, ROCK2, PKD and p-PKD were detected and GAPDH was used as a control. (b) Normal human keratinocytes were treated with different dosage LPA as indicated for 60min, nuclear protein were purified as described in *Materials and Methods*, western-blotting was performed to detect protein expression as indicated. (c) Normal human keratinocytes were stimulated with LPA for 12hrs, following pre-treated with LPAR5 inhibitor or vehicle for 2 hrs, RNA was isolated and qRT-PCR analysis was performed to determine the mRNA expression of psoriasis-associated inflammatory factors as indicated. Data are representative of three independent experiments .Data are presented as the mean±SD. P-values were determined using two-way ANOVA; *, P < 0.01; **, P < 0.001; ***, P < 0.0001.

Figure.S9.

**
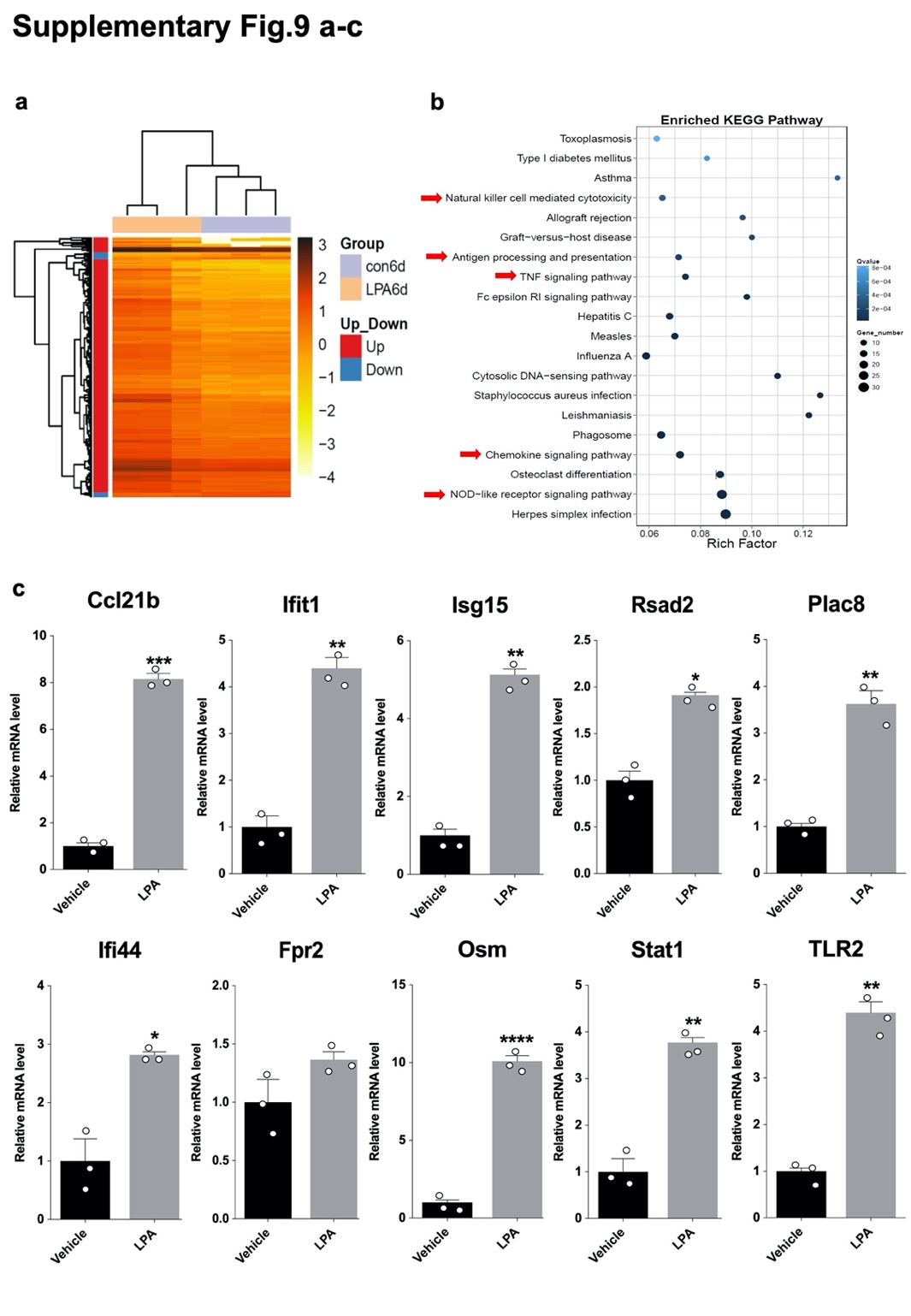
**

**RNA-Seq analysis the effect of LPA on gene expression profiles.** The ear skin of mice(n=3) were treated with IMQ plus LPA or vehicle for six consecutive days as same as Fig.1C. RNA was extracted from ear skin and gene expression profiles in this two groups were screened by high-throughput sequencing as described in *Materials and Methods*. (a)The heat maps of gene expression in control group and LPA group were summarized. The relative gene expression is described according to the color code shown below the cluster. RNA-seq was performed and DESeq2 was used to analyze differentially expressed genes. (b)The bubble chart showed the first 20 (KEGG) pathways in the encyclopedia of Kyoto genes and genomes that are being enriched. (c) RNA was isolated from ear skin of mice treated with IMQ or IMQ plus LPA and qRT-PCR analysis was performed to determine indicated mRNA expression. Data are presented as the mean±SD(n=3). P-values were determined using two-way ANOVA; *, P < 0.01; **, P < 0.001; ***, P < 0.0001.

Figure.S10.

**
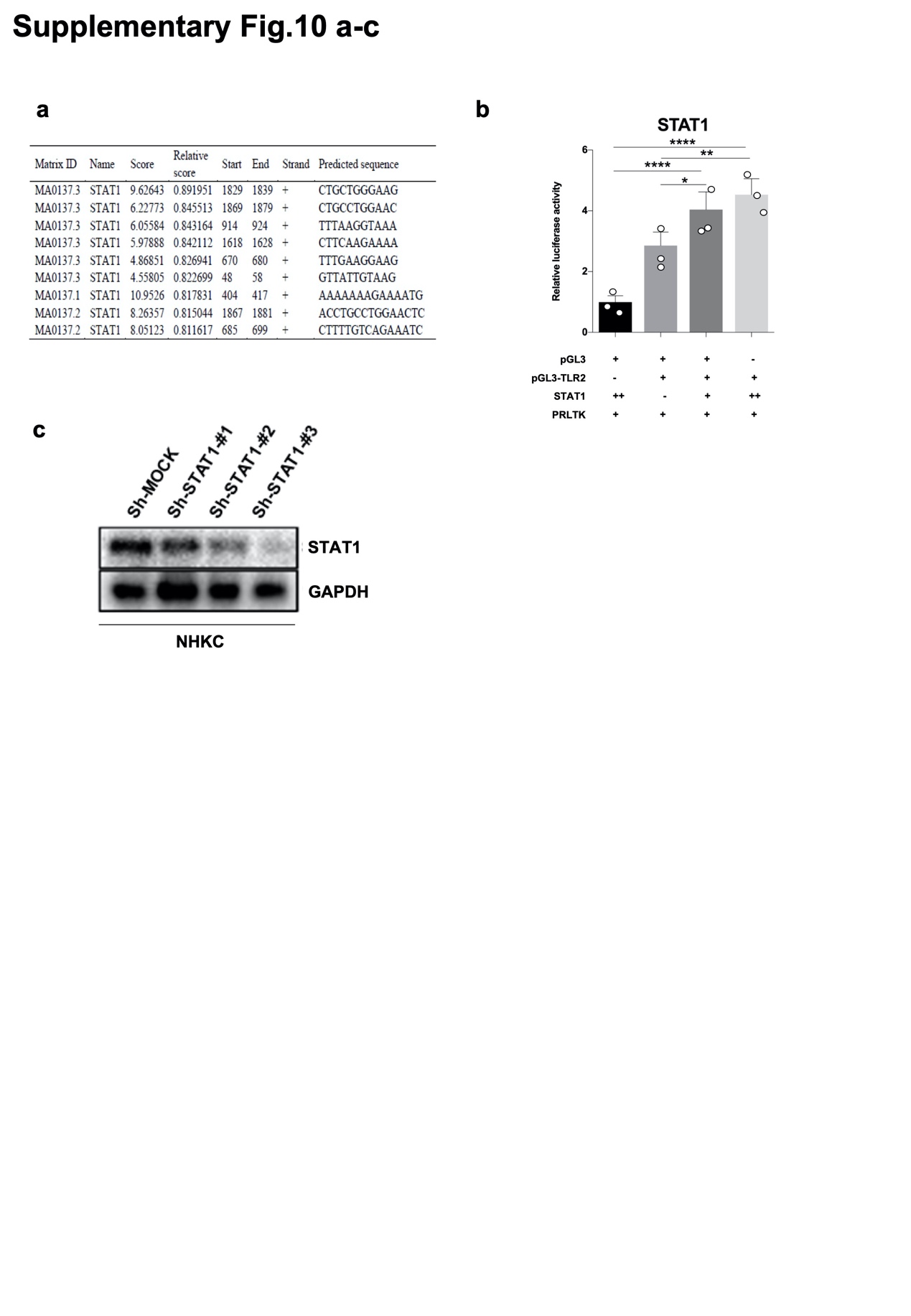
**

**Figure.S10.LPA induces TLR2 expression by activating STAT1.** (a) JASPAR software predicted multiple binding sites for TLR2 gene promoters recognized by Stat1. (b) The TLR2 luciferase reporter gene was generated as described in *Materials and Methods*. PGL3-TLR2 and Stat1 were transfected into 293T cells and double luciferase reporter gene assay was performed as described in *Materials and Methods*. Data from multiple experiments are expressed as the means ± S.D. Significant differences were evaluated using two-way ANOVA, *n=4, p<0.05. (c)Whole cell lysate of NHKC infected by lentiviral particles (sh-Mock, shSTAT1#-1, shSTAT1#-2 and shSTAT1#-3) were extracted and subjected to western blot analysis using antibodies to STAT1 as described in *Materials and Methods*, GAPDH was used as control.

Table S1.

**Supplemental Table 1. Basic clinical features of psoriasis patients and healthy volunteers**

|  |  | **Psoriasis patients (%)**  (n=120) |  | **Healthy volunteers (%)**  (n=100) | ***p*-value** |
| --- | --- | --- | --- | --- | --- |
| **Gender** | Male | 70 (58.3%) |  | 55 (55%) | 0.6191 |
|  | Female | 50 (41.7%) |  | 45 (45%) |  |
| **Age (year)** |  | 41.73±1.340 |  | 46.23±1.873 | 0.0555 |
| **BMI** |  | 24.26±0.379 |  | 23.10±0.307 | 0.0223 |

Table S2.

**Supplemental Table 2. LPA-mediated inflammatory response-related pathways（Top 10）**

| **#** | **Pathway** | **DEGs** | **Pvalue** | **Qvalue** |
| --- | --- | --- | --- | --- |
| 1 | NOD-like receptor signaling pathway | 28 | 1.502136e-14 | 1.269305e-12 |
| 2 | Chemokine signaling pathway | 18 | 2.2606e-08 | 9.551035e-07 |
| 3 | TNF signaling pathway | 12 | 3.890416e-06 | 5.057541e-05 |
| 4 | **Antigen processing and presentation** | 11 | 1.393373e-05 | 1.682000e-04 |
| 5 | Natural killer cell mediated cytotoxicity | 11 | 3.315901e-05 | 3.296396e-04 |
| 6 | **NF-kappa B signaling pathway** | 7 | 0.002070428 | 1.295935e-02 |
| 7 | RIG-I-like receptor signaling pathway | 6 | 002317227 | 1.398612e-02 |
| 8 | Cytokine-cytokine receptor interaction | 12 | 0.00269775 | 1.519733e-02 |
| 9 | **Toll-like receptor signaling pathway** | 7 | 0.006814092 | 3.112383e-02 |
| 10 | IL-17 signaling pathway | 5 | 0.03815556 | 1.334085e-01 |

Table S3.

**Supplemental Table 3. Detection of differentially expressed genes induced by LPA by RNA-seq（Top 30）**

| Gene Symbol | Fold change  LPA/Control | Gene Symbol | Fold change  LPA/Control |
| --- | --- | --- | --- |
| Ccl21b | 27.92 | Oasl1 | 6.71 |
| Zbp1 | 12.11 | Phf11b | 6.61 |
| Oasl2 | 11.88 | Ms4a4c | 6.51 |
| Oas1g | 11.32 | Gbp3 | 6.47 |
| Ifit1 | 10.72 | Ifi47 | 6.43 |
| Fcgr4 | 10.24 | Batf2 | 6.30 |
| Tgtp1 | 10.12 | Apol9b | 5.93 |
| Isg15 | 9.41 | Oas1a | 5.88 |
| Rsad2 | 8.95 | B430306N03Rik | 5.76 |
| Rtp4 | 8.88 | Oas2 | 5.70 |
| Plac8 | 8.05 | Pyhin1 | 5.62 |
| Ifi44 | 7.74 | Gm12250 | 5.62 |
| Fpr2 | 7.32 | Ccl5 | 5.52 |
| Iigp1 | 7.21 | Phf11d | 5.49 |
| Gm4951 | 7.00 | Ubd | 5.44 |

Table S4.

**Supplemental Table 4. Primer sequence of partial genes**

| **Primer** | **Forward (5’-3’)** | **Reverse (5’-3’)** |
| --- | --- | --- |
| CXCL1(h) | TCCTGCATCCCCCATAGTTA | CTTCAGGAACAGCCACCAGT |
| CXCL1(m) | GACTCCAGCCACACTCCAAC | TGACAGCGCAGCTCATTG |
| CXCL2(h) | CCCATGGTTAAGAAAATCATCG | CTTCAGGAACAGCCACCAAT |
| CXCL2(m) | AAAATCATCCAAAAGATACTGAACAA | CTTTGGTTCTTCCGTTGAGG |
| GAPDH(h) | CTCTGCTCCTCCTGTTCGAC | GCCCAATACGACCAAATCC |
| GAPDH(m) | ATGGTGAAGGTCGGTGTGA | AATCTCCACTTTGCCACTGC |
| IL-17A(h) | TGGGAAGACCTCATTGGTGT | GGATTTCGTGGGATTGTGAT |
| IL-17A(m) | CAGGGAGAGCTTCATCTGTGT | GCTGAGCTTTGAGGGATGAT |
| IL-17C(h) | GGTGTTGCACCTTGACAGACG | CGCCAACTGATCTGTGAGC |
| IL-17C(m) | CCTCTAGCTGGAACACAGTGC | GCGGTTCTCATCTGTGTCG |
| IL-6(h) | ATGGATGCTTCCAATCTG | CTGGCTTGTTCCTCACTAC |
| IL-6(m) | GCTACCAAACTGGATATAATCAGGA | CCAGGTAGCTATGGTACTCCAGAA |
| Lpar5(h) | CACTTGGTGGTCTACAGCTTG | GCGTAGTAGGAGAGACGAACG |
| Lpar5(m) | ACCTGGACATGATGTTTGCCA | GAGACCAGTCGCCAATACCA |
| S100A8(h) | GCCAAGCCTAACCGCTATAA | ATGATGCCCACGGACTTG |
| S100A8(m) | TCCTTGCGATGGTGATAAAA | GGCCAGAAGCTCTGCTACTC |
| S100A9(h) | GTGCGAAAAGATCTGCAAAA | TCAGCTGCTTGTCTGCATTT |
| S100A9(m) | GACACCCTGACACCCTGAG | TGAGGGCTTCATTTCTCTTCTC |
| STAT1(h) | CCATCCTTTGGTACAACATGC | TGCACATGGTGGAGTCAGG |
| STAT1(m) | TCACAGTGGTTCGAGCTTCAG | GCAAACGAGACATCATAGGCA |
| TNF-α(h) | CAGCCTCTTCTCCTTCCTGAT | GCCAGAGGGCTGATTAGAGA |
| TNF-α(m) | CTGTAGCCCACGTCGTAGC | TTGAGATCCATGCCGTTG |
| VEGF(h) | CGCAAGAAATCCCGGTATAA | AAATGCTTTCTCCGCTCTGA |
| VEGF(m) | AAAAACGAAAGCGCAAGAAA | TTTCTCCGCTCTGAACAAGG |
| TLR2(h) | AAGCAGCATATTTTACTGCTGG | CCTGAAACAAACTTTCATCGGT |
| TLR2(m) | CTCTTCAGCAAACGCTGTTCT | GGCGTCTCCCTCTATTGTATTG |
| Osm(m) | ATGCAGACACGGCTTCTAAGA | TTGGAGCAGCCACGATTGG |
| Ccl21b(m) | AGGCAGTGATGGAGGGGGA | GCTTAGAGTGCTTCCGGGGTA |
| Ifit1(m) | GCCTATCGCCAAGATTTAGATGA | TTCTGGATTTAACCGGACAGC |
| Isg15(m) | GGTGTCCGTGACTAACTCCAT | CTGTACCACTAGCATCACTGTG |
| Rsad2(m) | AGCATTAGGGTGGCTAGATCC | CTGAGTGCTGTTCCCATCTTC |
| Plac8(m) | CACCAACAGTTATCGTGACTCA | CCACACAGACAACACTCATTCA |
| Ifi44(m) | ATGCTCCAACTGACTGCTCG | ACAGCAATGCCTCTTGTCTTT |
| Fpr2(m) | CCGTCCTTTACGAGTCCTTACA | CAGGAGGTGAAGTAGAACTGGT |
